# The Long-Term Effects of Stress on Partner Weight Characteristics

**DOI:** 10.1371/journal.pone.0066353

**Published:** 2013-06-26

**Authors:** Jason M. Fletcher, Nathan Tefft

**Affiliations:** 1 Department of Health Policy and Management, Yale School of Public Health, New Haven, Connecticut, United States of America; 2 Department of Health Services, School of Public Health, University of Washington, Seattle, Washington, United States of America; Chiba University Center for Forensic Mental Health, Japan

## Abstract

**Background:**

Recent experimental evidence suggests that stressed males find heavier women more attractive than non-stressed males. The aim of this study is to examine whether these results also appear in actual mating patterns of adults from a national sample.

**Methods:**

Regression analysis linking partner weight measures to own measures of childhood stress, as measured by mistreatment. Cross-sectional data from the National Longitudinal Study of Adolescent Health, Romantic Partners Sample is used to measure partner weight, childhood stressful events, and socio-demographic characteristics. Childhood experiences of adult mistreatment are retrospectively collected.

**Results:**

Men who experienced childhood mistreatment are more likely to have obese female partners during young adulthood. The results are strongest for interactions with social services, adult neglect and physical abuse. We also present novel evidence of the opposite association in similarly stressed women whose male partners are more likely to be thin.

**Conclusions:**

These results suggest that preferences for partner characteristics are sensitive to histories of stress and that previously hypothesized patterns occur outside the experimental setting.

## Introduction

There is a substantial body of evidence supporting an inverse association between resource scarcity and body size preference based on the hypothesis that a larger body size may indicate greater access to food or other resources [1]. This hypothesis has been generalized to motivate studies relating several forms of stress to body size preferences, much of which has been focused on studying men’s preferences for women’s body size and type. For example, hunger [2], psychological stress [1], socioeconomic status [3], and financial insecurity [4], [5] have been suggested as potential manifestations of resource insecurity affecting men’s preferences, generally revealing a pattern that men under greater stress prefer heavier women.

The relationship between resource scarcity or stress and women’s preferences for men’s body types is less clear, with some evidence suggesting that hungry women exhibit a slightly higher preference for heavier, taller, and older men [2]. The paucity of conclusive evidence for women may be partly explained by women reporting personality characteristics as relatively more important than body type than do men [6]. In addition, women could express partner preferences for body type along dimensions that may be less immediately affected by resource scarcity such as height [7] or waist-chest ratio [8]. Under the “dual mating hypothesis”, which proposes that women’s sexual preferences change across the menstrual cycle [9], a greater variability in women’s preferences could lead to greater variability in pairing across physical characteristics. Furthermore, there is evidence that women’s stated preferences about body type are less correlated with their partner’s actual characteristics than are men’s stated preferences [10]. This may suggest that body mass index, specifically, is less important in partner decisions for women than it is for men.

The long-term effects of stress on preferences for partner characteristics remain largely unknown. Child abuse is known to be associated with a variety of long-term health-related behaviors and outcomes including depression, substance abuse, and suicide [11]–[13], any of which may affect partner choice. Evidence based on “betrayal trauma theory” suggests that child abuse can have a direct effect on partner choice when trauma leads to dissociation and a lack of betrayal awareness by leading a victim of abuse to ignore signals about potential partner betrayal. Consistent with this possibility, participants in a study who experienced high trauma rated partner loyalty as less important than participants who experienced medium or low trauma [14].

The “cycle of violence” hypothesis also predicts that violence through child abuse or other trauma will increase the likelihood of violent acts later in life, and stress has been found to increase aggression more acutely for persons with a history of child maltreatment in an experimental setting [15]. A primary assumption underlying the theory of marriage and intra-household bargaining [16] is that partners select and maintain a relationship only if the relationship is valued more by each partner than is the value of being single. An implication of this assumption is that relationships which involve negative impacts on a partner’s well-being, such as violence toward that partner, may involve a lower “threat point” to leave the relationship. Partners who have what are considered more desirable characteristics, e.g. thinness in women [10], may be therefore less likely to join or more likely to end a relationship involving violence. In a large cross-sectional survey, women with any lifetime experiences of intimate partner violence (IPV) reported a significantly higher prevalence of elevated body mass index (BMI) than women who had not experienced IPV, but there was no significant difference for men across IPV status [17].

Studies that have tested the relationship between stress and sex-related or partner preferences for body weight typically only measure surveyed preferences and induce stress in an experimental setting. To our knowledge, no study examines the relationship between reported long term stress and the body type profiles of men and women who self-report as partners. By utilizing a unique, nationally representative data set of partner dyads we are able to study the relationship between early life stressors, such as abuse, experienced by each partner pair and their effects on partner body weight characteristics in real world relationships.

## Methods

### Ethical Statement

The human subjects committee at Yale University approved this study. Participants provided written informed consent to participate in the Add Health.

### Participants

Participants include nearly 1,500 pairs of individuals in the “Couples Sample” collected in the third wave (2000/1) of the National Longitudinal Study of Adolescent Health (Add Heath). Of these individuals, approximately 500 pairs were married, 500 pairs were cohabitating, and 500 pairs were dating. The Add Health is a nationally representative study of 7^th^ to 12^th^ graders from 1994 and 1995 who have been followed longitudinally. Approximately 750 individuals from the main study were sub-sampled and a “romantic partner” was recruited for the Couples Sample. Each individual in the pairs were administered a similar survey. Participants ranged in age between 18–43 (the main Add Health respondents ranged in age between 18–26, while their partners’ ages were unrestricted). Each individual’s body mass index was measured; the male average was 27.05 and the female average was 26.41.

### Materials

The Add Health survey included standard socio-demographic questions, including age, gender, race/ethnicity, maternal educational attainment, and own educational attainment as well as retrospective reports of childhood mistreatment, which will be our key measures of long term stress. All information is known from each individual in each pair, allowing a dyadic analysis of current partners. Key measures for the analysis include each partner’s weight, history of childhood mistreatment, and gender.

#### Childhood mistreatment measures

Respondents were asked a series of retrospective questions about their parents and other adults’ mistreatment before the respondent was in 6^th^ grade. These include: how often they were (i) left alone when an adult should have been with them (ii) not taken care of in their basic needs, such as keeping clean and providing food and clothing (iii) slapped, hit, or kicked (iv) touched in a sexual way (v) had social services investigated the family or tried to take them out of their living situation. Categorical answers for (i-iv) included: (A) one time (B) two times (C) three to five times (D) six to ten times (E) more than ten times (F) this never happened. Question (v) allowed any number to be reported (range 0 to 60). A small proportion of individuals refused to answer these questions or responded they “don’t know”. We combine these responses in the main analysis by using a factor analysis on items (i-iv) [13] and using item (v) as a summary measure of abuse that was severe enough to be reported to social services, which occurred in approximately 6 percent of the sample. In auxiliary analysis we also create “ever measures” of i-iv, where approximately 40% were “left alone”, 10–15% had “unmet basic needs”, 30% were physically abused, and approximately 5% were sexually abused (see Table S1A).

#### Weight measures

Respondents weights and heights were measured by interviewers. Body mass index is created by using the ratio of weight (kilograms) and height (meters- squared). Overweight status is defined as a BMI greater or equal to 25 and obese status is defined as a BMI greater or equal to 30.

### Statistical Analysis

Recent experimental evidence has suggested that, for males, induced stress increases preferences for larger body size [1]. The current analysis extends this evidence by using OLS regression analysis to estimate associations between partner weight and own childhood mistreatment. This is accomplished with the following empirical specification:
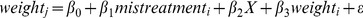
(1)where the vector *X* is a set of dyadic controls (e.g. age, education, maternal education, race/ethnicity) and 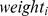 controls the processes that generate homophily in partner characteristics among couples. As described above, we use three alternative measures of “mistreatment” when estimating equation (1)–(A) a principal component summary of our child mistreatment measures (B) the social services indicator and (C) each of the principal component measures as separate predictors. Equation (1) is estimated separately for men and women (with respect to individual *i*). All analysis was completed using Stata Version 11.0.

## Results

Table S1 provides sample descriptive statistics and detailed information on the variables described above and studied in the analyses. Table 1 presents results from equation (1) where the outcome is female weight and the key independent variable is male childhood mistreatment. Each column reports a separate regression and unstandardized beta coefficients are presented for each independent variable. Column 1 shows that males with higher measures of childhood mistreatment are 3.2 percentage points more likely to have a female partner who is obese. Column 2 shows that males who were investigated by social services during childhood are 9 percentage points more likely to have an obese female partner. Columns 3–6 show that these results are qualitatively similar for outcomes of female BMI and female overweight, though these results are not statistically significant.

**Table 1:** The Effects of Childhood Abuse in Males on Adult Partner Weight Status.

Outcome	Female Obese	Female Obese	Female BMI	Female BMI	Female Overweight	Female Overweight
Abuse Indicator	MistreatmentFactor	SocialServices	MistreatmentFactor	SocialServices	MistreatmentFactor	SocialServices
Male Abuse Measure	0.032*	0.090*	0.454**	0.736	0.030	0.054
	(0.016)	(0.054)	(0.223)	(0.775)	(0.022)	(0.060)
Female Abuse Measure	0.026	−0.045	0.396	−0.493	0.030	−0.008
	(0.019)	(0.048)	(0.299)	(0.710)	(0.021)	(0.059)
Male Weight Status	0.166***	0.169***	0.269***	0.258***	0.148***	0.126***
	(0.034)	(0.032)	(0.042)	(0.041)	(0.032)	(0.029)
Female Age	0.015**	0.013**	0.166*	0.167*	0.009	0.012*
	(0.007)	(0.006)	(0.100)	(0.097)	(0.008)	(0.007)
Male Age	0.004	0.006	0.052	0.075	0.012**	0.012***
	(0.005)	(0.005)	(0.068)	(0.074)	(0.005)	(0.005)
Male Maternal Education	−0.011***	−0.007*	−0.195***	−0.128**	−0.014**	−0.011*
	(0.004)	(0.004)	(0.061)	(0.064)	(0.005)	(0.006)
Female Maternal Education	−0.004	−0.009**	−0.112	−0.167**	−0.017***	−0.019***
	(0.005)	(0.004)	(0.072)	(0.069)	(0.006)	(0.005)
Male Black	0.033	0.019	0.266	−0.092	−0.027	−0.034
	(0.071)	(0.063)	(1.033)	(0.974)	(0.068)	(0.063)
Female Black	0.076	0.070	2.062**	2.126**	0.155**	0.141**
	(0.066)	(0.058)	(0.985)	(0.977)	(0.060)	(0.059)
Married Couple	0.095***	0.090***	1.848***	1.699***	0.110***	0.106***
	(0.034)	(0.029)	(0.442)	(0.397)	(0.028)	(0.028)
Observations	1141	1189	1141	1189	1141	1189
R-squared	0.082	0.079	0.120	0.113	0.089	0.080

Standard errors in parentheses.

*** p<0.01,

** p<0.05,

* p<0.1.

Additional Controls: Constant, Indicator for Hispanic Male/Female.

Table 2 examines more specific measures of childhood mistreatment by including four separate measures of whether the individual was ever “left alone”, had “unmet basic needs”, was ever physically abused, and was ever sexually abused. Column 1 suggests that childhood experiences with unmet needs and physical abuse are positively related to female partner BMI during adulthood. Columns 2 and 3 suggests an increase in the likelihood of having an overweight female partner for those with unmet basic needs and an increase in the likelihood of having an obese female partner for those experiencing childhood physical abuse. No significant relationships are found for being left alone or sexually abused.

**Table 2:** The Effects of Childhood Abuse in Males on Adult Partner Weight Status: Abuse Categories.

Outcome	FemaleBMI	Female Overweight	Female Obese
Male Left Alone	−0.506	−0.012	−0.016
	(0.448)	(0.035)	(0.037)
Male Unmet Basic Needs	1.281**	0.095**	0.069
	(0.613)	(0.044)	(0.047)
Male Physical Abuse	0.905**	0.039	0.055*
	(0.446)	(0.031)	(0.033)
Male Sexual Abuse	−0.913	−0.076	−0.037
	(1.036)	(0.070)	(0.066)
Male Weight	0.270***	0.146***	0.167***
	(0.042)	(0.033)	(0.033)
Female Age	0.187*	0.011	0.016**
	(0.103)	(0.008)	(0.007)
Male Age	0.043	0.012**	0.004
	(0.068)	(0.005)	(0.005)
Male Maternal Education	−0.185***	−0.013**	−0.011**
	(0.062)	(0.005)	(0.004)
Female Maternal Education	−0.119	−0.017***	−0.005
	(0.073)	(0.006)	(0.005)
Male Black	0.214	−0.028	0.025
	(1.007)	(0.068)	(0.069)
Female Black	2.104**	0.159**	0.085
	(0.979)	(0.061)	(0.066)
Male Hispanic	−0.215	−0.012	−0.020
	(0.722)	(0.060)	(0.049)
Female Hispanic	0.374	0.070	0.002
	(0.744)	(0.061)	(0.051)
Married Couple	1.962***	0.119***	0.102***
	(0.440)	(0.028)	(0.034)
Female Left Alone	1.133**	0.112***	0.064**
	(0.437)	(0.031)	(0.028)
Female Basic Needs	−0.747	−0.079*	−0.029
	(0.557)	(0.045)	(0.039)
Female Physical Abuse	−0.238	−0.021	−0.050*
	(0.384)	(0.035)	(0.030)
Female Sexual Abuse	0.927	0.080	0.086*
	(0.723)	(0.060)	(0.044)
Observations	1141	1141	1141
R-squared	0.133	0.105	0.093

Standard errors in parentheses.

*** p<0.01,

** p<0.05,

* p<0.1.

Additional Controls: Constant.

While the prior literature typically focuses on the effects of stress on men in determining partner weight, we next examine the converse–what is the effect of long-term stress (as measured by childhood mistreatment) on women in determining (male) partner weight? Table 3 repeats Table 1 specifications but transposes the gender of the partner–now we ask the relationship between women’s exposure to childhood mistreatment and the weight attributes of their adult male partners. Unlike the results for the effects of stressful male childhoods, the results suggest that females who experience stressful childhoods are less likely to have partners who are overweight or obese. For example, in Column 2 in Table 3, we find that females who were investigated by social services during childhood are nearly 12 percentage points less likely to have an obese romantic partner as an adult than women who report no such investigation. Likewise, Columns 3 and 4 show strong negative correlations between the mistreatment factor and male partner BMI as well as between the social services indicator and male partner BMI (1.5 units lower vs women not investigated by social services). Columns 5 and 6 again shows that women who experienced childhood mistreatment are between 5–10 percentage points less likely to have a romantic partner who is overweight than women who did not have this experience. Unlike our results in Table 2, Table 4 shows little consistent evidence for the “type” of stressor that is related to partner weight. Only one of the specific childhood mistreatment variables are statistically related to male partner weight.

**Table 3:** The Effects of Childhood Abuse in Females on Adult Partner Weight Status.

Outcome	Male Obese	Male Obese	Male BMI	Male BMI	Male Overweight	Male Overweight
Abuse Indicator	MistreatmentFactor	SocialServices	MistreatmentFactor	SocialServices	MistreatmentFactor	SocialServices
Male Abuse Measure	0.028*	−0.032	0.296	−0.624	0.028	−0.068
	(0.016)	(0.046)	(0.213)	(0.606)	(0.022)	(0.060)
Female Abuse Measure	−0.016	−0.117***	−0.494**	−1.467**	−0.054**	−0.100*
	(0.019)	(0.039)	(0.212)	(0.572)	(0.021)	(0.053)
Female Weight Status	0.182***	0.183***	0.188***	0.187***	0.148***	0.125***
	(0.037)	(0.035)	(0.028)	(0.029)	(0.031)	(0.029)
Female Age	0.004	0.001	0.094	0.068	0.007	0.006
	(0.006)	(0.005)	(0.086)	(0.074)	(0.008)	(0.007)
Male Age	0.008**	0.008**	0.130***	0.133***	0.011**	0.012**
	(0.004)	(0.004)	(0.046)	(0.045)	(0.005)	(0.005)
Male Maternal Education	0.000	0.002	0.043	0.058	0.007	0.010*
	(0.007)	(0.006)	(0.069)	(0.066)	(0.006)	(0.006)
Female Maternal Education	−0.002	−0.003	−0.035	−0.037	−0.001	−0.002
	(0.004)	(0.004)	(0.062)	(0.063)	(0.006)	(0.006)
Male Black	−0.060	−0.042	−1.044	−0.671	−0.090	−0.078
	(0.060)	(0.055)	(0.690)	(0.657)	(0.061)	(0.057)
Female Black	0.068	0.068	1.380*	1.309*	0.137**	0.167***
	(0.064)	(0.058)	(0.760)	(0.694)	(0.067)	(0.057)
Married Couple	0.075***	0.058**	0.775**	0.605*	0.103***	0.081**
	(0.026)	(0.023)	(0.348)	(0.334)	(0.033)	(0.032)
Observations	1141	1189	1141	1189	1141	1189
R-squared	0.057	0.060	0.094	0.094	0.067	0.063

Standard errors in parentheses.

*** p<0.01,

** p<0.05,

* p<0.1.

Additional Controls: Constant, Indicator for Hispanic Male/Female.

**Table 4:** The Effects of Childhood Abuse in Females on Adult Partner Weight Status: Abuse Categories.

Outcome	MaleBMI	Male Overweight	Male Obese
Female Left Alone	−0.563	−0.033	−0.022
	(0.368)	(0.030)	(0.031)
Female Unmet Basic Needs	−0.665	−0.103*	−0.060
	(0.497)	(0.052)	(0.048)
Female Physical Abuse	0.067	−0.010	0.037
	(0.357)	(0.033)	(0.028)
Female Sexual Abuse	−0.516	−0.024	−0.031
	(0.603)	(0.058)	(0.046)
Female Weight	0.191***	0.148***	0.184***
	(0.028)	(0.032)	(0.037)
Female Age	0.084	0.006	0.004
	(0.090)	(0.008)	(0.006)
Male Age	0.134***	0.011**	0.008**
	(0.048)	(0.005)	(0.004)
Male Maternal Education	0.044	0.007	0.000
	(0.071)	(0.006)	(0.007)
Female Maternal Education	−0.041	−0.002	−0.003
	(0.061)	(0.006)	(0.004)
Male Black	−0.931	−0.079	−0.048
	(0.692)	(0.062)	(0.058)
Female Black	1.281*	0.130*	0.057
	(0.755)	(0.068)	(0.062)
Male Hispanic	0.531	0.052	0.017
	(0.716)	(0.062)	(0.051)
Female Hispanic	0.491	0.066	0.032
	(0.600)	(0.059)	(0.046)
Married Couple	0.752**	0.101***	0.075***
	(0.351)	(0.034)	(0.026)
Male Left Alone	0.532	0.043	0.033
	(0.342)	(0.027)	(0.033)
Male Unmet Basic Needs	0.004	0.038	0.011
	(0.466)	(0.048)	(0.040)
Male Physical Abuse	−0.105	−0.017	−0.004
	(0.396)	(0.036)	(0.030)
Male Sexual Abuse	0.823	−0.050	0.066
	(0.934)	(0.073)	(0.073)
Observations	1141	1141	1141
R-squared	0.097	0.070	0.061

Standard errors in parentheses.

*** p<0.01,

** p<0.05,

* p<0.1.

Additional Controls: Constant.

## Discussion

In this study, we examined the long-term effects of childhood stress, as measured by mistreatment, on preferences for body type. We used a nationally representative sample of adults first interviewed as 7^th^ to 12^th^ graders in 1994 and 1995 who were later interviewed along with their romantic partners in 2001. We compared partner weight characteristics for both men and women, including BMI, obese, and overweight status, among respondents according to their experience of childhood mistreatment after controlling for several potential confounders including own weight status, age, education, race, ethnicity, and marital status. Broadly speaking, the results suggest that men who experienced child abuse tend to prefer a heavier female body size in their partners while women who experienced child abuse may prefer lighter male body size.

More specifically, men who experienced a high mistreatment factor according to a factor analysis of questions describing several aspects of parental and other adult abuse experiences had female partners with significantly greater BMI and were significantly more likely to be in a relationship with an obese female partner. Men whose abuse was severe enough to be reported to social services similarly had partners with greater BMI and who were more likely to be obese, although only the latter effect was statistically significant. When simultaneously comparing potential child abuse experiences in a regression analysis, men whose basic needs were not taken care of and men who were physically abused as children were those likely to have heavier partners.

Unlike many previous studies, we also considered how child mistreatment among women affects their preferences for male body type according to the weight and abuse measures described above. Perhaps unexpectedly, women with a higher mistreatment factor had male partners with lower average BMI and probability of being overweight, and women whose mistreatment was reported to social services exhibited preferences for lighter males, according to every measure. When considering all of the underlying types of child mistreatment simultaneously the results were largely insignificant but the point estimates for the partner weight preference effect was consistently negative, in almost every case.

Overall these results in part confirm previous work on body type preference but also suggest opportunities for future research. In particular, male preferences for romantic partners appear consistent with the “environmental security hypothesis” [1], [2] in the long term, as measured by the effects of childhood stress on adult partner preference. Further supporting this confirmatory evidence is the finding that the strongest association between childhood mistreatment and female body size arose for men who identified their needs as not having been met as children. In this sense, prolonged experiences of unmet need may have similar long run effects as immediate sensations indicating scarcity. That men who experienced physical abuse as children similarly exhibited a preference for heavier body types also lends support to the hypothesis that the “cycle of violence” effect [15] may lead those men to enter into relationships in which partner violence is more accepted.

Perhaps the most unexpected results were for the effects of childhood mistreatment on partner body size preferences among women. As described earlier, women tended to have partners with lower BMI and who were less likely to be overweight or obese if they experienced child abuse. Interestingly, this relationship does not appear to be concentrated among any subset of mistreatment types but is similar for women who were left alone, whose needs were unmet, and who were physically or sexually abused. A potential explanation for this association may lie in the fact that BMI and, by implication overweight and obesity status, do not adequately distinguish between muscle mass and body fat when measuring the underlying weight component [18]. Since the sample used in the current analysis does not include other measures of body type we are not able to separately distinguish alternative potential explanations for female preferences for body type, such as waist-chest ratio [8]. A more complete classification of body types may yield important evidence in future research in this area.

An important strength of this study is that it is based on a nationally representative sample, so the findings herein may be broadly generalized. In addition, this analysis offers to our knowledge the first “real world” study of actual partner pairings in a generalizable sample. Previous research in this area has typically examined stated preferences for hypothetical ideal types, often in experimental settings. As such the findings from this study may be particularly relevant for considering how child abuse and related circumstances including poverty and resource deprivation among men and women may affect the desirability of body types, both self-perceived and perceived among others.

The primary limitations of this study result from common tradeoffs encountered when conducting observational studies, more generally. At the expense of producing novel findings with respect to “real world” partner choices, the current study may not adequately control for all relevant characteristics of the partner dyads. For example, it has been hypothesized that stress may affect “self-esteem, empathy, or related constructs” [1] and it is indirectly through these channels, rather than directly, that stress influences partner weight preferences. Although we confirmed that our results are generally robust to the inclusion of other known outcomes of childhood mistreatment including depression and substance abuse (see Tables S2 and S3), more research is needed. This general concern may be more relevant for women than men since the results for the latter are broadly consistent with previous hypotheses and experiments, but it is not possible to know with certainty. Additionally, our measure of childhood mistreatment is retrospective and could possibly be affected by reporting bias. We believe that any reporting bias would be unrelated to weight preferences and would have difficulty explaining the gender differences in weight preferences we report. However, future research able to access mistreatment (or other stressors) prospectively would be a useful next step. Future research leveraging natural experiments or quasi-experimental designs may also more accurately estimate causal relationships in “real world” settings.

## Supporting Information

Table S1 **Sample Descriptive Statistics.**
(DOCX)

Table S2 **The Effects of Childhood Abuse in Males on Adult Partner Weight Status: With Behavioral Controls.**
(DOCX)

Table S3 **The Effects of Childhood Abuse in Females on Adult Partner Weight Status: With Behavioral Controls.**
(DOCX)

## References

[1] Swami V, Tovée MJ (2012) The impact of psychological stress on men’s judgements of female body size. PloS one 7: e42593. Available: http://dx.plos.org/10.1371/journal.pone.0042593. Accessed 24 February 2013. 10.1371/journal.pone.0042593 PMC3414440 22905153

[2] Pettijohn TFI, Sacco DJ, Yerkes M (2009) Hungry people prefer more mature mates: a field test of the environmental security hypothesis. The Journal of Social, Evolutionary, and Cultural Psychology 3: 216–232.

[3] Swami V, Frederick DA, Aavik T, Alcalay L, Allik J, et al (2010) The Attractive Female Body Weight and Female Body Dissatisfaction in 26 Countries Across 10 World Regions: Results of the International Body Project I. Personality and Social Psychology Bulletin. 36 309–325 Available: http://psp.sagepub.com/content/36/3/309.abstract. 10.1177/0146167209359702 20179313

[4] Nelson LD, Morrison EL (2005) The Symptoms of Resource Scarcity: Judgments of Food and Finances Influence Preferences for Potential Partners. Psychological Science 16 167–173 Available: http://pss.sagepub.com/content/16/2/167.abstract. 15686584 10.1111/j.0956-7976.2005.00798.x

[5] Swami V, Tovée MJ, Furnham A (2008) Does financial security influence judgements of female physical attractiveness? Journal of Socio-Economics 37: 1363–1370.

[6] Braun MF, Bryan A (2006) Female waist-to-hip and male waist-to-shoulder ratios as determinants of romantic partner desirability. Journal of Social and Personal Relationships 23 805–819 Available: http://spr.sagepub.com/content/23/5/805.abstract.

[7] Pawlowski B, Jasienska G (2005) Women’s preferences for sexual dimorphism in height depend on menstrual cycle phase and expected duration of relationship. Biological Psychology 70: 38–43. 16038772 10.1016/j.biopsycho.2005.02.002

[8] Maisey DS, Vale ELE, Cornelissen PL, Tovée MJ (1999) Characteristics of male attractiveness for women. The Lancet 353: 1500 Available: http://www.sciencedirect.com/science/article/pii/S0140673699004389. 10.1016/s0140-6736(99)00438-9 10232328

[9] Larson CM, Haselton MG, Gildersleeve KA, Pillsworth EG (2013) Changes in women’s feelings about their romantic relationships across the ovulatory cycle. Hormones and Behavior 63: 128–135 Available: http://www.sciencedirect.com/science/article/pii/S0018506X12002486. 23085495 10.1016/j.yhbeh.2012.10.005

[10] Courtiol A, Picq S, Godelle B, Raymond M, Ferdy J-B (2010) From preferred to actual mate characteristics: the case of human body shape. PloS one 5: e13010. Available: http://dx.plos.org/10.1371/journal.pone.0013010. Accessed 27 February 2013. 10.1371/journal.pone.0013010 PMC2946385 20885953

[11] Neigh GN, Gillespie CF, Nemeroff CB (2009) The Neurobiological Toll of Child Abuse and Neglect. Trauma, Violence, & Abuse 10 389–410 Available: http://tva.sagepub.com/content/10/4/389.abstract. 10.1177/1524838009339758 PMC6492037 19661133

[12] Buckingham ET, Daniolos P (2013) Longitudinal Outcomes for Victims of Child Abuse. Current Psychiatry Reports 15: 1–7 Available: http://dx.doi.org/10.1007/s11920-012-0342-3. 10.1007/s11920-012-0342-3 23307564

[13] Fletcher JM (2009) Childhood mistreatment and adolescent and young adult depression. Social Science & Medicine 68: 799–806 Available: http://www.sciencedirect.com/science/article/pii/S0277953608006527. 19155114 10.1016/j.socscimed.2008.12.005

[14] Gobin RL (2011) Partner Preferences Among Survivors of Betrayal Trauma. Journal of Trauma & Dissociation 13: 152–174 Available: http://dx.doi.org/10.1080/15299732.2012.642752. 10.1080/15299732.2012.642752 22375805

[15] Gowin J, Green C, Alcorn III J, Swann A, Moeller FG, et al. (2013) The role of cortisol and psychopathy in the cycle of violence. Psychopharmacology: 1–12. Available: http://dx.doi.org/10.1007/s00213-013-2992-1. 10.1007/s00213-013-2992-1 PMC5481784 23371492

[16] Becker GS (1974) A Theory of Marriage. Economics of the family: Marriage, children, and human capital. UMI. 299–351.

[17] Breiding MJ, Black MC, Ryan GW (2008) Chronic Disease and Health Risk Behaviors Associated with Intimate Partner Violence–18 U.S. States/Territories, 2005. Annals of Epidemiology 18: 538–544 Available: http://www.sciencedirect.com/science/article/pii/S1047279708000483. 18495490 10.1016/j.annepidem.2008.02.005

[18] Burkhauser R V, Cawley J (2008) Beyond BMI: The value of more accurate measures of fatness and obesity in social science research. Journal of Health Economics 27: 519–529 Available: http://www.sciencedirect.com/science/article/pii/S0167629607001130. 18166236 10.1016/j.jhealeco.2007.05.005

