# Supplementary material for: The Long-Term Effects of Stress on Partner Weight Characteristics
Source: PLoS One. 2013 Jun 26;8(6):e66353. doi: 10.1371/journal.pone.0066353 (PMC3694100; doi:10.1371/journal.pone.0066353)
Supplement: Table S1 — Sample Descriptive Statistics. (DOCX) [file pone.0066353.s001.docx]

Table S1

Sample Descriptive Statistics

| Variable | Obs | Mean | Std Dev | Min | Max |
| --- | --- | --- | --- | --- | --- |
| Female BMI | 1399 | 26.41 | 6.61 | 15.6 | 51.8 |
| Male BMI | 1399 | 27.05 | 5.50 | 13.1 | 53.0 |
| Female Obese (0/1) | 1399 | 0.24 | 0.43 | 0 | 1 |
| Male Obese (0/1) | 1399 | 0.26 | 0.44 | 0 | 1 |
| Female Overweight (0/1) | 1399 | 0.48 | 0.50 | 0 | 1 |
| Male Overweight (0/1) | 1399 | 0.59 | 0.49 | 0 | 1 |
| Male Social Services (0/1) | 1294 | 0.06 | 0.24 | 0 | 1 |
| Female Social Services (0/1) | 1304 | 0.07 | 0.25 | 0 | 1 |
| Male Left Alone (0/1) | 1301 | 0.44 | 0.50 | 0 | 1 |
| Male Unmet Basic Needs(0/1) | 1319 | 0.15 | 0.36 | 0 | 1 |
| Male Physical Abuse(0/1) | 1325 | 0.32 | 0.47 | 0 | 1 |
| Male Sexual Abuse (0/1) | 1331 | 0.04 | 0.20 | 0 | 1 |
| Female Left Alone (0/1) | 1330 | 0.39 | 0.49 | 0 | 1 |
| Female Unmet Basic Needs(0/1) | 1365 | 0.09 | 0.29 | 0 | 1 |
| Female Physical Abuse(0/1) | 1361 | 0.28 | 0.45 | 0 | 1 |
| Female Sexual Abuse (0/1) | 1360 | 0.07 | 0.25 | 0 | 1 |
| Male Mistreatment Index | 1242 | 0.02 | 0.70 | -0.5 | 3.9 |
| Female Mistreatment Index | 1298 | -0.03 | 0.67 | -0.5 | 4.5 |
| Male Binge (0/1) | 1388 | 0.52 | 0.50 | 0 | 1 |
| Female Binge (0/1) | 1394 | 0.33 | 0.47 | 0 | 1 |
| Male Number of Cigarettes | 1389 | 5.11 | 9.85 | 0 | 100 |
| Female Number of Cigarettes | 1395 | 3.59 | 7.62 | 0 | 100 |
| Male Depression Scale | 1392 | 4.10 | 3.76 | 0 | 25 |
| Female Depression Scale | 1395 | 5.28 | 4.39 | 0 | 24 |
| Female Age | 1399 | 21.81 | 2.37 | 18 | 40 |
| Male Age | 1399 | 23.41 | 3.25 | 18 | 43 |
| Male Maternal Education | 1384 | 12.60 | 2.52 | 0 | 17 |
| Female Maternal Education | 1392 | 12.84 | 2.45 | 0 | 17 |
| Male Black (0/1) | 1399 | 0.18 | 0.39 | 0 | 1 |
| Female Black (0/1) | 1399 | 0.17 | 0.37 | 0 | 1 |
| Male Hispanic (0/1) | 1399 | 0.15 | 0.36 | 0 | 1 |
| Female Hispanic (0/1) | 1399 | 0.14 | 0.35 | 0 | 1 |
| Married Couple Indicator (0/1) | 1399 | 0.34 | 0.47 | 0 | 1 |
| Male White (0/1) | 1399 | 0.60 | 0.49 | 0 | 1 |
| Female White(0/1) | 1399 | 0.62 | 0.48 | 0 | 1 |

Notes:

*BMI* (body mass index) is the ratio of weight (kilograms) and height (meters-squared). *Obese* is BMI greater than or equal to 30, *Overweight* is BMI greater than or equal to 25.

*Social Services* is a binary variable indicating at least one instance based on the following question: “By the time you started 6^th^ grade, how often had Social Services investigated how you were taken care of or tried to take you out of your living situation?

*Left Alone* is a binary variable indicating at least one instance based on the following question: By the time you started 6th grade, how often had your parents or other adult care-givers left you home alone when an adult should have been with you?

*Unmet Basic Needs* is a binary variable indicating at least one instance based on the following question: By the time you started 6th grade how often had your parents or other adult care-givers not taken care of your basic needs, such as keeping you clean or providing food or clothing?

*Physical abuse* is a binary variable indicating at least one instance based on the following question: By the time you started 6th grade, how often had your parents or other adult care-givers slapped, hit, or kicked you?

*Sexual abuse* is a binary variable indicating at least one instance based on the following question: By the time you started 6th grade how often had one of your parents or other adult care-givers touched you in a sexual way, forced you to touch him or her in a sexual way , or forced you to have sexual relations?

*Mistreatment indices* are the first factor of a principle components analysis of the following variables: “left alone”, “unmet basic needs”, “physical abuse”, and “sexual abuse”.

*Binge* is a binary variable indicating whether the respondent reported any days of binge drinking (5+ drinks in a sitting) in the past 12 months.

*Number of Cigarettes* is reported number of cigarettes smoked per day (on days when smoking cigarettes) in the past 30 days

*Depression Scale* is the summed CESD score from the 9-item scale in Wave 3

*Age* is reported age in years at Wave 3

*Maternal Education* is the reported category of education for the mother or mother figure of the respondent. We code 8^th^ grade or less (8 years), more than 8^th^ grade, but did not graduate from high school (11), went to a business or vocation school instead of high school (12), high school graduate (12), completed a GED (12), went to business or trade school after high school (13), went to college but did not graduate (14), graduated from college (16), professional training beyond a four year college (17), never went to school (0).

*Married Couple indicator* is equal to 1 if the couple is married and zero if the couple is dating or cohabiting.
