# Supplementary material for: The Long-Term Effects of Stress on Partner Weight Characteristics
Source: PLoS One. 2013 Jun 26;8(6):e66353. doi: 10.1371/journal.pone.0066353 (PMC3694100; doi:10.1371/journal.pone.0066353)
Supplement: Table S2 — The Effects of Childhood Abuse in Males on Adult Partner Weight Status: With Behavioral Controls. (DOCX) [file pone.0066353.s002.docx]

Table S2

The Effects of Childhood Abuse in Males on Adult Partner Weight Status: With Behavioral Controls

| Outcome | Female Obese | Female Obese | Female Obese | Female Overweight | Female Overweight | Female Overweight | Female  BMI | Female BMI | Female  BMI |
| --- | --- | --- | --- | --- | --- | --- | --- | --- | --- |
| Abuse Indicator | Factor | Social Services | Categories | Factor | Social Services | Categories | Factor | Social Services | Categories |
| Male Abuse Measure | 0.034** | 0.075 |  | 0.032 | 0.044 |  | 0.490** | 0.560 |  |
|  | (0.017) | (0.053) |  | (0.022) | (0.058) |  | (0.229) | (0.746) |  |
| Female Abuse Measure | 0.017 | -0.056 |  | 0.020 | -0.016 |  | 0.262 | -0.657 |  |
|  | (0.020) | (0.047) |  | (0.022) | (0.057) |  | (0.306) | (0.703) |  |
| Male Weight Status | 0.177*** | 0.176*** | 0.177*** | 0.155*** | 0.132*** | 0.152*** | 0.286*** | 0.268*** | 0.287*** |
|  | (0.034) | (0.032) | (0.033) | (0.033) | (0.029) | (0.033) | (0.042) | (0.041) | (0.042) |
| Female Age | 0.013* | 0.011* | 0.015* | 0.008 | 0.013* | 0.011 | 0.148 | 0.162 | 0.174 |
|  | (0.007) | (0.006) | (0.007) | (0.008) | (0.007) | (0.009) | (0.107) | (0.103) | (0.111) |
| Male Age | 0.004 | 0.007 | 0.004 | 0.011** | 0.011** | 0.011** | 0.053 | 0.074 | 0.042 |
|  | (0.005) | (0.006) | (0.005) | (0.005) | (0.005) | (0.005) | (0.070) | (0.079) | (0.070) |
| Male Maternal Education | -0.009** | -0.006 | -0.009** | -0.013** | -0.011** | -0.013** | -0.165*** | -0.111* | -0.158** |
|  | (0.004) | (0.004) | (0.004) | (0.005) | (0.005) | (0.005) | (0.060) | (0.062) | (0.061) |
| Female Maternal Education | -0.002 | -0.007 | -0.003 | -0.015*** | -0.018*** | -0.016*** | -0.087 | -0.150** | -0.099 |
|  | (0.005) | (0.004) | (0.005) | (0.006) | (0.006) | (0.006) | (0.073) | (0.070) | (0.075) |
| Male Black | 0.047 | 0.036 | 0.040 | -0.017 | -0.039 | -0.017 | 0.662 | 0.296 | 0.637 |
|  | (0.071) | (0.065) | (0.070) | (0.068) | (0.063) | (0.069) | (0.924) | (0.906) | (0.901) |
| Female Black | 0.053 | 0.058 | 0.060 | 0.144** | 0.154** | 0.144** | 1.431* | 1.680* | 1.413 |
|  | (0.067) | (0.059) | (0.067) | (0.062) | (0.062) | (0.063) | (0.863) | (0.904) | (0.858) |
| Male Hispanic | -0.007 | -0.005 | -0.007 | -0.007 | 0.005 | -0.011 | -0.016 | 0.120 | -0.041 |
|  | (0.052) | (0.044) | (0.050) | (0.060) | (0.055) | (0.058) | (0.744) | (0.732) | (0.704) |
| Female Hispanic | 0.007 | 0.010 | 0.004 | 0.081 | 0.058 | 0.073 | 0.405 | 0.197 | 0.280 |
|  | (0.052) | (0.058) | (0.053) | (0.059) | (0.061) | (0.060) | (0.788) | (0.803) | (0.791) |
| Married Couple | 0.097*** | 0.091*** | 0.103*** | 0.113*** | 0.105*** | 0.119*** | 1.859*** | 1.703*** | 1.954*** |
|  | (0.035) | (0.029) | (0.035) | (0.029) | (0.029) | (0.029) | (0.464) | (0.411) | (0.465) |
| Male Left Alone |  |  | -0.010 |  |  | -0.006 |  |  | -0.476 |
|  |  |  | (0.038) |  |  | (0.036) |  |  | (0.446) |
| Male Unmet Basic Needs |  |  | 0.072 |  |  | 0.103** |  |  | 1.380** |
|  |  |  | (0.046) |  |  | (0.044) |  |  | (0.617) |
| Male Physical Abuse |  |  | 0.058* |  |  | 0.039 |  |  | 0.970** |
|  |  |  | (0.033) |  |  | (0.031) |  |  | (0.453) |
| Male Sexual Abuse |  |  | -0.046 |  |  | -0.075 |  |  | -1.086 |
|  |  |  | (0.068) |  |  | (0.072) |  |  | (1.071) |
| Female Left Alone |  |  | 0.063** |  |  | 0.112*** |  |  | 1.148*** |
|  |  |  | (0.028) |  |  | (0.031) |  |  | (0.425) |
| Female Basic Needs |  |  | -0.041 |  |  | -0.089* |  |  | -0.965 |
|  |  |  | (0.041) |  |  | (0.045) |  |  | (0.584) |
| Female Physical Abuse |  |  | -0.051* |  |  | -0.020 |  |  | -0.221 |
|  |  |  | (0.030) |  |  | (0.037) |  |  | (0.392) |
| Female Sexual Abuse |  |  | 0.074 |  |  | 0.066 |  |  | 0.734 |
|  |  |  | (0.045) |  |  | (0.062) |  |  | (0.747) |
| Male Binge Drink | -0.040* | -0.033 | -0.040* | -0.025 | -0.040 | -0.026 | -0.428 | -0.491 | -0.421 |
|  | (0.024) | (0.025) | (0.023) | (0.030) | (0.029) | (0.029) | (0.426) | (0.384) | (0.416) |
| Female Binge Drink | -0.012 | -0.011 | -0.013 | -0.005 | -0.006 | -0.013 | -0.413 | -0.284 | -0.486 |
|  | (0.026) | (0.031) | (0.026) | (0.029) | (0.029) | (0.030) | (0.326) | (0.371) | (0.332) |
| Male Number of Cigarettes | -0.000 | 0.001 | -0.001 | 0.002 | 0.002 | 0.002 | 0.007 | 0.016 | 0.003 |
|  | (0.002) | (0.002) | (0.002) | (0.002) | (0.001) | (0.002) | (0.022) | (0.021) | (0.023) |
| Female Number of Cigarettes | 0.005** | 0.004* | 0.005** | 0.002 | 0.004 | 0.002 | 0.050* | 0.042 | 0.049* |
|  | (0.002) | (0.002) | (0.002) | (0.002) | (0.002) | (0.002) | (0.030) | (0.031) | (0.029) |
| Male Depression Scale | 0.001 | 0.003 | 0.001 | -0.002 | 0.001 | -0.003 | 0.009 | 0.022 | -0.001 |
|  | (0.004) | (0.004) | (0.004) | (0.004) | (0.004) | (0.004) | (0.057) | (0.055) | (0.054) |
| Female Depression Scale | 0.005 | 0.003 | 0.005* | 0.005 | -0.000 | 0.004 | 0.096* | 0.059 | 0.097* |
|  | (0.003) | (0.003) | (0.003) | (0.004) | (0.004) | (0.004) | (0.052) | (0.055) | (0.051) |
| Observations | 1121 | 1160 | 1121 | 1121 | 1160 | 1121 | 1121 | 1160 | 1121 |
| R-squared | 0.095 | 0.090 | 0.106 | 0.094 | 0.088 | 0.110 | 0.132 | 0.124 | 0.146 |

Standard errors in parentheses. *** p<0.01, ** p<0.05, * p<0.1. Additional Controls: Constant
