# Supplementary material for: The Long-Term Effects of Stress on Partner Weight Characteristics
Source: PLoS One. 2013 Jun 26;8(6):e66353. doi: 10.1371/journal.pone.0066353 (PMC3694100; doi:10.1371/journal.pone.0066353)
Supplement: Table S3 — The Effects of Childhood Abuse in Females on Adult Partner Weight Status: With Behavioral Controls. (DOCX) [file pone.0066353.s003.docx]

Table S3

The Effects of Childhood Abuse in Females on Adult Partner Weight Status: With Behavioral Controls

| Outcome | Male Obese | Male Obese | Male Obese | Male Overweight | Male Overweight | Male Overweight | Male BMI | Male BMI | Male BMI |
| --- | --- | --- | --- | --- | --- | --- | --- | --- | --- |
| Abuse Indicator | Factor | Social Services | Categories | Factor | Social Services | Categories | Factor | Social Services | Categories |
| Male Abuse Measure | 0.033** | -0.029 |  | 0.031 | -0.064 |  | 0.319 | -0.532 |  |
|  | (0.016) | (0.046) |  | (0.022) | (0.061) |  | (0.214) | (0.621) |  |
| Female Abuse Measure | -0.010 | -0.116*** |  | -0.047** | -0.096* |  | -0.365 | -1.414** |  |
|  | (0.020) | (0.039) |  | (0.021) | (0.052) |  | (0.221) | (0.562) |  |
| Female Weight Status | 0.193*** | 0.193*** | 0.196*** | 0.154*** | 0.131*** | 0.154*** | 0.201*** | 0.196*** | 0.204*** |
|  | (0.037) | (0.035) | (0.037) | (0.032) | (0.029) | (0.033) | (0.027) | (0.029) | (0.027) |
| Female Age | 0.007 | 0.003 | 0.006 | 0.011 | 0.009 | 0.011 | 0.131 | 0.099 | 0.120 |
|  | (0.006) | (0.005) | (0.006) | (0.007) | (0.007) | (0.007) | (0.087) | (0.075) | (0.091) |
| Male Age | 0.006* | 0.006 | 0.006* | 0.010** | 0.010** | 0.010** | 0.113*** | 0.113*** | 0.117*** |
|  | (0.004) | (0.004) | (0.004) | (0.005) | (0.005) | (0.005) | (0.043) | (0.042) | (0.044) |
| Male Maternal Education | -0.002 | 0.001 | -0.001 | 0.005 | 0.008 | 0.005 | 0.006 | 0.022 | 0.010 |
|  | (0.007) | (0.007) | (0.007) | (0.006) | (0.006) | (0.006) | (0.067) | (0.068) | (0.069) |
| Female Maternal Education | -0.003 | -0.004 | -0.004 | -0.003 | -0.002 | -0.004 | -0.068 | -0.050 | -0.072 |
|  | (0.004) | (0.004) | (0.004) | (0.006) | (0.006) | (0.006) | (0.062) | (0.066) | (0.062) |
| Male Black | -0.063 | -0.042 | -0.051 | -0.082 | -0.067 | -0.073 | -1.121 | -0.654 | -1.037 |
|  | (0.061) | (0.057) | (0.059) | (0.065) | (0.062) | (0.065) | (0.708) | (0.696) | (0.709) |
| Female Black | 0.080 | 0.086 | 0.068 | 0.128* | 0.156** | 0.120* | 1.480* | 1.423* | 1.394* |
|  | (0.066) | (0.063) | (0.064) | (0.071) | (0.063) | (0.072) | (0.811) | (0.780) | (0.804) |
| Male Hispanic | 0.007 | 0.072 | 0.010 | 0.041 | 0.073 | 0.040 | 0.369 | 1.102 | 0.402 |
|  | (0.053) | (0.055) | (0.051) | (0.062) | (0.063) | (0.063) | (0.724) | (0.735) | (0.714) |
| Female Hispanic | 0.030 | 0.013 | 0.024 | 0.058 | 0.079 | 0.053 | 0.400 | 0.324 | 0.370 |
|  | (0.047) | (0.049) | (0.047) | (0.062) | (0.058) | (0.062) | (0.602) | (0.557) | (0.610) |
| Married Couple | 0.075*** | 0.061** | 0.076*** | 0.100*** | 0.078** | 0.098*** | 0.741** | 0.614* | 0.728** |
|  | (0.028) | (0.024) | (0.028) | (0.032) | (0.033) | (0.033) | (0.359) | (0.336) | (0.360) |
| Male Left Alone |  |  | 0.031 |  |  | 0.030 |  |  | 0.417 |
|  |  |  | (0.033) |  |  | (0.028) |  |  | (0.338) |
| Male Unmet Basic Needs |  |  | 0.018 |  |  | 0.048 |  |  | 0.080 |
|  |  |  | (0.041) |  |  | (0.046) |  |  | (0.456) |
| Male Physical Abuse |  |  | -0.002 |  |  | -0.014 |  |  | -0.093 |
|  |  |  | (0.030) |  |  | (0.037) |  |  | (0.402) |
| Male Sexual Abuse |  |  | 0.057 |  |  | -0.063 |  |  | 0.841 |
|  |  |  | (0.071) |  |  | (0.072) |  |  | (0.937) |
| Female Left Alone |  |  | -0.019 |  |  | -0.028 |  |  | -0.524 |
|  |  |  | (0.031) |  |  | (0.030) |  |  | (0.373) |
| Female Basic Needs |  |  | -0.052 |  |  | -0.097* |  |  | -0.490 |
|  |  |  | (0.048) |  |  | (0.052) |  |  | (0.501) |
| Female Physical Abuse |  |  | 0.039 |  |  | -0.011 |  |  | 0.065 |
|  |  |  | (0.029) |  |  | (0.033) |  |  | (0.365) |
| Female Sexual Abuse |  |  | -0.028 |  |  | -0.014 |  |  | -0.376 |
|  |  |  | (0.045) |  |  | (0.057) |  |  | (0.585) |
| Male Binge Drink | 0.028 | 0.026 | 0.028 | 0.045 | 0.037 | 0.044 | 0.503 | 0.432 | 0.501 |
|  | (0.024) | (0.024) | (0.024) | (0.030) | (0.030) | (0.030) | (0.312) | (0.309) | (0.315) |
| Female Binge Drink | 0.024 | 0.018 | 0.022 | 0.015 | 0.002 | 0.013 | 0.460 | 0.343 | 0.476 |
|  | (0.028) | (0.028) | (0.027) | (0.033) | (0.031) | (0.033) | (0.329) | (0.337) | (0.327) |
| Male Number of Cigarettes | -0.000 | 0.000 | -0.000 | -0.003** | -0.001 | -0.003** | -0.023 | 0.002 | -0.022 |
|  | (0.001) | (0.001) | (0.001) | (0.001) | (0.002) | (0.001) | (0.018) | (0.021) | (0.018) |
| Female Number of Cigarettes | -0.002 | -0.001 | -0.002 | -0.000 | -0.001 | -0.000 | -0.035 | -0.032 | -0.035 |
|  | (0.002) | (0.002) | (0.002) | (0.003) | (0.002) | (0.003) | (0.028) | (0.027) | (0.027) |
| Male Depression Scale | -0.003 | -0.004 | -0.003 | 0.002 | 0.000 | 0.003 | -0.002 | -0.013 | -0.000 |
|  | (0.003) | (0.003) | (0.003) | (0.004) | (0.004) | (0.005) | (0.044) | (0.046) | (0.042) |
| Female Depression Scale | -0.002 | -0.001 | -0.002 | -0.003 | -0.001 | -0.003 | -0.063* | -0.041 | -0.059* |
|  | (0.003) | (0.002) | (0.003) | (0.004) | (0.004) | (0.004) | (0.033) | (0.031) | (0.033) |
| Observations | 1121 | 1160 | 1121 | 1121 | 1160 | 1121 | 1121 | 1160 | 1121 |
| R-squared | 0.065 | 0.064 | 0.067 | 0.073 | 0.063 | 0.076 | 0.108 | 0.100 | 0.111 |

Standard errors in parentheses. *** p<0.01, ** p<0.05, * p<0.1. Additional Controls: Constant
